# The use of alpha-adrenergic antagonists in pediatric nephrolithiasis: a systematic review

**DOI:** 10.3389/fped.2024.1396659

**Published:** 2024-12-02

**Authors:** Firas Haddad, Walid A. Farhat, Shannon Cannon

**Affiliations:** ^1^ Faculty of Medicine, American University of Beirut, Beirut, Lebanon; ^2^ Divison of Pediatric Urology, Department of Urology, University of Wisconsin School of Medicine and Public Health, Madison, WI, United States

**Keywords:** nephrolithiasis, Pediatric Urology, systematic review, alpha blockers, stone disease

## Abstract

**Objective:**

To evaluate existing clinical evidence for the efficacy of alpha blockers in the management of pediatric stone disease.

**Methods:**

We conducted a systematic review following Preferred Reporting Items for Systematic Reviews and Meta-Analyses (PRISMA) guidelines. Cohort and randomized control trials of patients less than 18 years old with kidney stones managed with alpha-adrenergic antagonists were included. Outcomes included stone expulsion time, stone passage rate, mean number of pain episodes, and mean need for analgesics. We performed data extraction of the selected articles, and results were assimilated and synthesized qualitatively. Data extraction and risk of bias assessment was conducted by two independent reviewers.

**Results:**

Of 257 relevant studies, 9 studies with 1,039 patients were included. Six studies measured stone expulsion time, with 5 studies noting statistically significant decreases in stone expulsion time for the treatment group compared to the control. Seven studies measured the stone expulsion rate, and 5 reported a statistically significant increased expulsion rate in the treatment group. Four studies reported a decrease in the mean number of pain episodes in the treatment group and two studies showed a decreased analgesic requirement compared to control. Two studies found alpha blockers not superior to watchful waiting after shock wave lithotripsy. Risk of bias was high in some studies, primarily due to incomplete reporting on methodology and study design.

**Conclusions:**

Alpha blockers are supported by a growing body of evidence to be effective against nephrolithiasis in children, however large-scale, well-designed studies are needed to confirm these findings.

**Systematic Review Registration:**

https://www.crd.york.ac.uk/prospero/display_record.php?RecordID=330068, PROSPERO (CRD42022330068).

## Introduction

Kidney stones in the pediatric population pose a significant public health concern, which has substantially increased over time. It is reported that there has been a five-fold increase in the incidence of pediatric urolithiasis in the past 20 years (1, 2). The standard care for such patients consists of non-invasive and minor surgical procedures to help pass the stones. Recent increase in the use of minimally invasive approaches and miniaturized technologies have been aided by dynamic technological advancements. Virtual reality, augmented reality, mixed reality, 3D modeling reconstructions have altered the landscape of the surgical management of pediatric nephrolithiasis. However, these advancements necessitate the physician to stay up to date with these advancements and are yet to become standard practice (3). To add, surgical procedures are not only costly, but also necessitate the use of anesthesia in the patients (4, 5). This has shed light on alternative interventions, such as pharmacotherapy, to aid with stone passage. Alpha-adrenergic antagonists, or alpha blockers, have gained significant attention, and there has been a rise of studies and trials that assess the use of alpha blockers in the expulsion of kidney stones in the adult population (6, 7). Further, alpha blockers have proven efficacious and safe in adult nephrolithiasis and are particularly helpful for passage of obstructing distal ureteral stones (8). Extensive review of alpha blocker use in adult stone disease has reinforced its utility, but the question remains of whether such drugs can help with the passage of stones in the pediatric population. Few clinical trials have assessed the use of alpha blockers in the pediatric patients, and prior efforts to synthesize existing data are either outdated or limited in scope (7, 8).

The purpose of this study is to conduct a systematic review of the randomized controlled trials and cohort studies that assess the use of alpha blockers in the passage of kidney stones in the pediatric population.

## Methods

### Eligibility criteria

This systematic review was done in accordance to the PRISMA guidelines (9). Randomized Controlled Trials (RCTs) and Cohort studies (both retrospective and prospective) were eligible for inclusion in the study. Articles were included if they met the following inclusion criteria: (1) studies were reported in English, (2) studies comparing the use of alpha blockers to placebo or to each other, (3) studies conducted in the pediatric population (age < 18), (4) studies measuring at least two of the following outcome measures were included: stone clearance rate, stone expulsion time, number of pain episodes, or mean need for analgesia. Studies that included pediatric patients in a larger cohort of adult patients were excluded. Studies that were reported in abstract form but for which the whole manuscript could not be obtained were also excluded. Meta-analysis was not performed due to expected heterogeneity of study designs, medications used for intervention, and outcomes reported in the included studies.

### Information sources and search strategy

The following databases were searched: Medline, PubMed, Cochrane, EMBASE, and Google Scholar. The search strategy was written by FFH, and the search was run on May 20, 2022 on all databases. Results were exported from each database and uploaded onto a citation manager (EndNote, Philadelphia, USA) where we then performed deduplication. Grey Literature was searched via World Health Organization International Clinical Trials Registry Platform Search Portal, ClinicalTrials.gov, and unpublished abstracts presented at scientific meetings of the following professional societies: American Urological Association, Societies for Pediatric Urology, American Academy of Pediatrics Section on Urology, Pediatric Academic Societies, European Association of Urology.

### Article screening

The resultant articles after deduplication were manually selected for inclusion by FFH and STSC. The articles were selected by the two in blind mode to minimize the biases that may arise, and any difference in judgement was discussed and resolved with a third judge (WAF). After the articles were selected, references of the selected articles were manually searched for any additional references that may have been missed in the search strategy.

### Data extraction

Data were extracted by FFH and STSC. The data extraction process was first calibrated, with both involved team members extracting data from the same two articles and comparing methods of extraction. Once alignment of extraction method was achieved, the remaining articles were divided between the two authors for independent extraction, while consulting WAF for any inquiries or confusion during the extraction process. The data were collected on the following variables and outcome measures: country, sample size, mean patient age, gender distribution, stone location, mean stone size, therapy, follow up time, duration of medication use, dosage, adverse effects, stone clearance rate, stone expulsion time, number of pain episodes, and mean need for analgesia.

### Risk of bias assessment

Risk of bias assessment was conducted by FFH and STSC, with any conflicting decisions resolved through WAF as a third assessment. Risk of Bias was conducted according to the Cochrane method, assessing for selection, performance, detection, attrition, reporting, and other biases. For each article, the presence of the following biases was assessed: selection, performance, detection, attrition, and reporting bias. Each bias was evaluated as low, high, or unclear. For cohort studies, the Newcastle Ottawa Scale was used for quality assessment in selection, comparability and outcome. In this score, selection bias can receive up to 4 points, comparability can receive up to 1 point, and outcome can receive up to 3 points based on criteria listed in the scale. Increased number of points is indicative of higher quality studies. To perform assessment, the first two articles were assessed for bias by the two authors for the purpose of process calibration. The remaining articles were divided among the evaluators and assessed independently, with WAF available for consultation.

## Results

**Figure 1:**
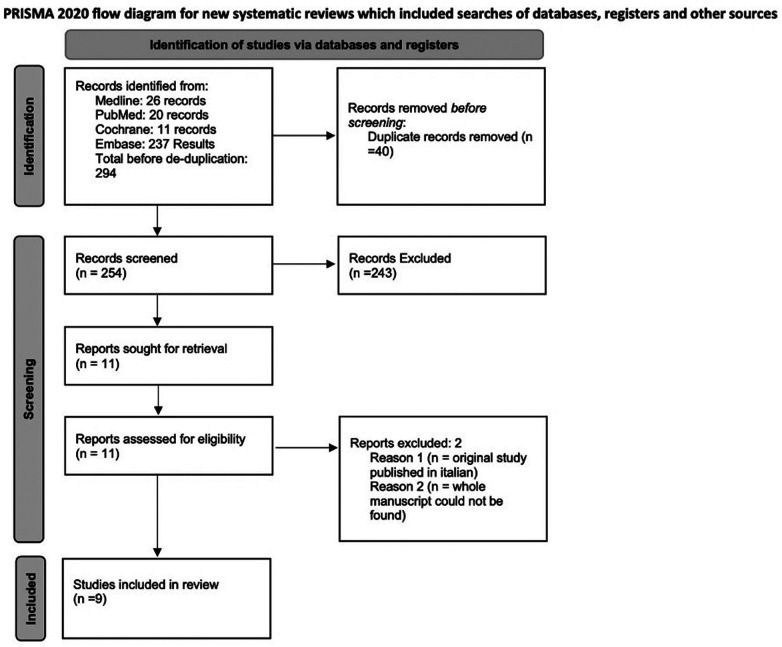
PRISMA flow diagram.

**Figure 2:**
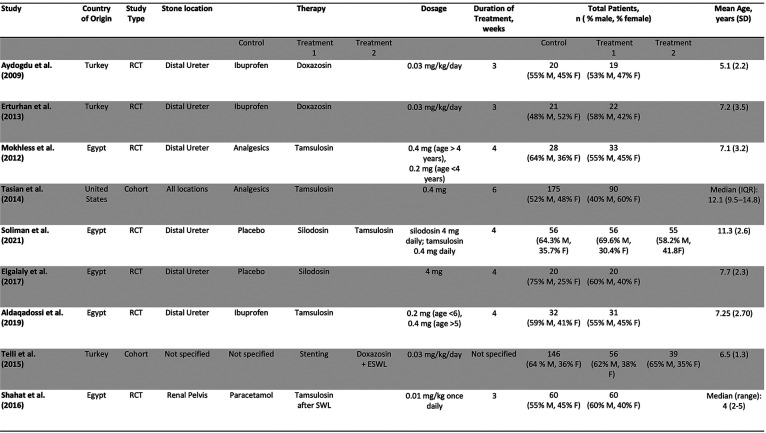
Baseline characteristics of included studies.

**Figure 3:**
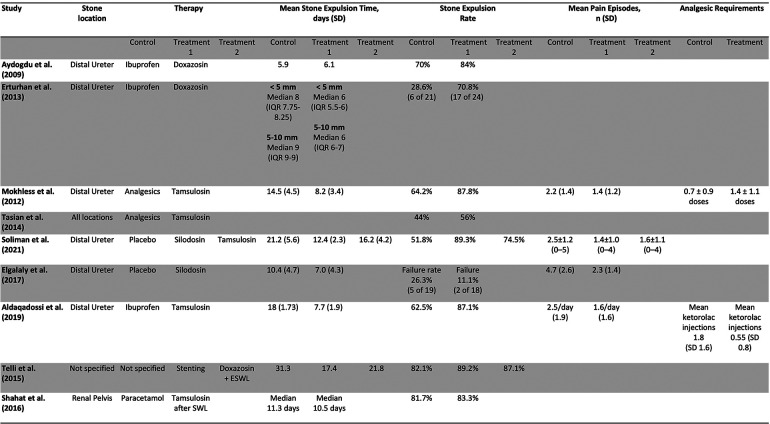
Outcomes of interest in included studies.

**Figure 4:**
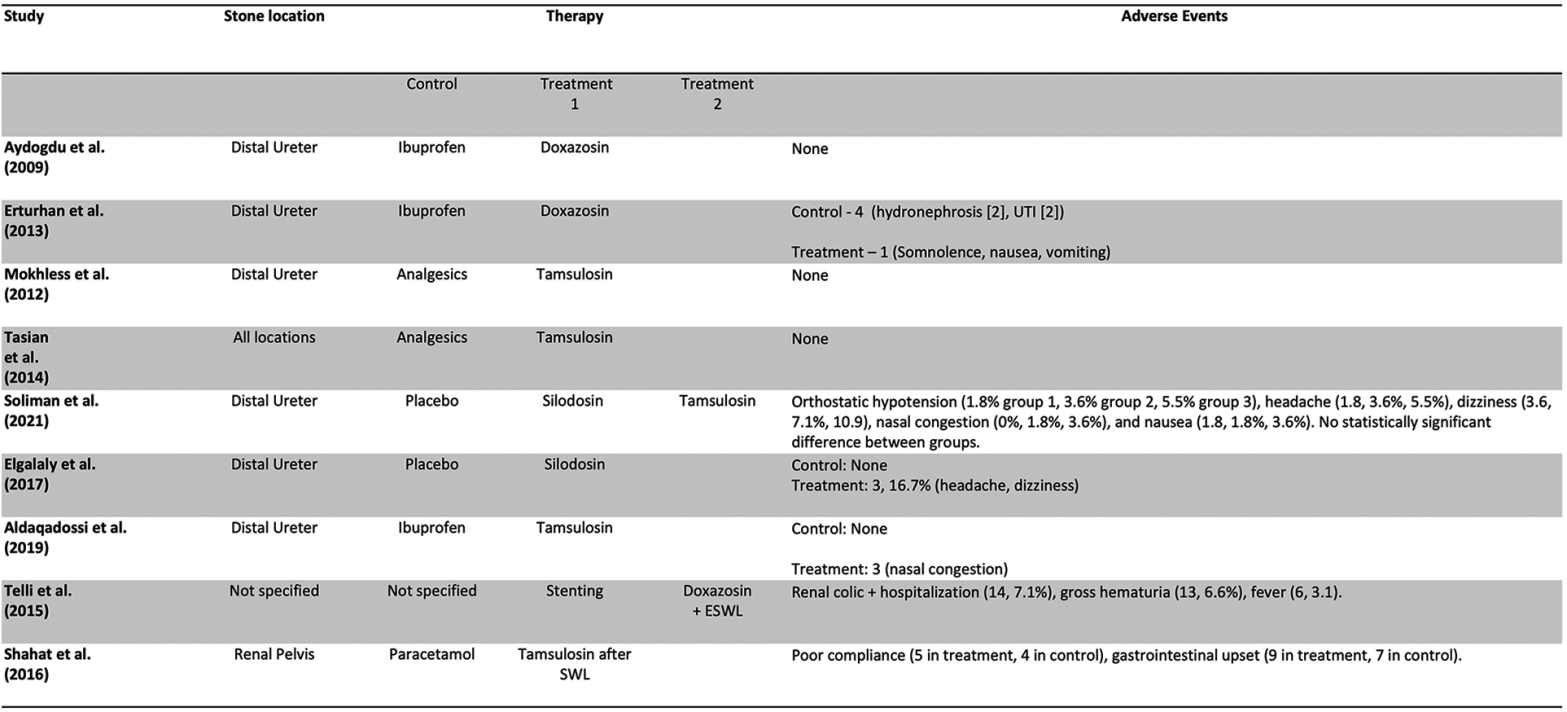
Adverse events reported in included studies.

Out of 257 Records retrieved after de-duplication, 9 studies, including 1,039 patients and 1 abstract met inclusion criteria and were included in the study. Figure 1 is a PRISMA flow diagram outlining the article selection process. Figures 2, 3 outline characteristics and outcome measures of the included studies. Five of the studies were conducted in Egypt, 3 in Turkey, and 1 in the United States. There were 7 RCTs and 2 retrospective cohort studies selected for inclusion. Three alpha blockers were the focus of studies included in this review: doxazosin, tamsulosin and silodosin. Tamsulosin was included in 5 studies, doxazosin in 3 studies, and silodosin in 2 studies. There was one 3-arm study in tamsulosin and silodosin were compared to placebo. Six studies focused on distal ureteral stones, while 1 study evaluated alpha blockers for use in stones in all ureteral locations. Two studies evaluated use of alpha blockers for renal stones after extracorporeal shockwave lithotripsy (SWL). In general, the interventions were well-tolerated with minimal side effects recorded. The adverse events are presented in Figure 4.

### Studies assessing the use of doxazosin

Three studies assessed the use of doxazosin in managing kidney stones in children (10–12). Two studies gave 0.03 mg/kg/day of doxazosin and ibuprofen 20 mg/kg/day for a period of 3 weeks and compared them to a control group taking ibuprofen alone (10, 11). A third study compared ureteral stenting, doxazosin, and watchful waiting to each other (12). All 3 studies assessed stone expulsion time. One of the studies found no statistically significant difference in stone expulsion time between the doxazosin group and the control group (10). Two studies found a decrease in stone expulsion time compared to control (11, 12). All 3 studies assessed the stone expulsion rate compared to control (9–11). Two studies found no statistically significant increase in stone expulsion rate compared to control (10, 12). One study found a statistically significant decrease in stone expulsion rate (11). One assessed stone expulsion rate by size and found that the group taking doxazosin passed stones <5 mm at higher rates than stones >5 mm (11). One study assessed the stone passage rate by age and found that children less than 6 years of age passed the stone at higher rates than their older counterpart (11). One study found no difference in the number of repeat SWL episodes after an initial SWL episode while taking doxazosin, watchful waiting, or patients with history of stent. This study found no difference in the number of SWL episodes (12).

### Studies assessing the use of tamsulosin

Four studies assessed the use of tamsulosin in managing pediatric kidney stones. The studies gave tamsulosin for periods varying from 3 to 6 weeks and gave varying doses of the drug (Figure 3) (13–16). All the studies compared tamsulosin to a control group taking standard analgesics such as ibuprofen, paracetamol, and ketorolac for intractable pain (12–15). One of the studies assessed the use of tamsulosin after SWL (16). Three studies assessed the stone passage time, and 2 of the studies found a statistically significant decrease in stone passage time between the two groups (13, 15). The third study found no significant difference in stone passage time after SWL between both groups (16). All 4 studies assessed stone passage rate. Three of the studies found a statistically significant increase in the stone passage rate in the tamsulosin group after the control (13, 15). The last study found no significant difference in stone passage rate after SWL when comparing tamsulosin to control (16). Two studies assessed the mean number of pain episodes, and both studies found a statistically significant decrease in pain episodes in the treatment group compared to control (13, 15). Two studies assessed the need for analgesia in the two groups, and both studies found a decrease in the need for analgesia in the treatment group compared to control (12, 14). One study assessed the stone passage by location and size, and found that stones at the UPJ or UVJ, and smaller stones were more likely to pass (14).

### Studies assessing the use of silodosin

Two studies assessed the use of silodosin. Both studies gave 4 mg of silodosin for a period of 4 weeks (17, 18). One study compared silodosin to a control group taking placebo (17). Another study had 3 groups, one taking silodosin 4 mg, one taking tamsulosin 0.4 mg, and one taking placebo (18). Both studies assessed the stone expulsion time. One study found a significant decrease in stone passage time between both groups (17). The other study found a statistically significant decrease in stone passage time between the group taking silodosin, and the two groups taking tamsulosin or placebo (18). The study also found a statistically significant decrease in stone passage time between the tamsulosin and the placebo group. Both studies assessed the stone passage rate. One of the studies found no statistically significant difference in stone passage rate between the two groups (17). The other study found a statistically significant difference in stone passage rate between the group taking silodosin, and the two groups taking tamsulosin or placebo. The study also found a statistically significant difference in stone passage rate between the tamsulosin and the placebo group (18). Both studies assessed the mean number of pain episodes. One study found a statistically significant decrease in pain episodes in the treatment group compared to control (17). The other study found that both the silodosin group and the tamsulosin had a significantly decreased number of pain episodes compared to control, and no significant difference between the silodosin and tamsulosin group (18).

### Efficacy of alpha blockers by stone location

Six studies assessed the use of alpha blockers in kidney stones in the lower ureter (10, 11, 13, 15, 17, 18). Two studies assessed the use of doxazosin, three studies assessed the use of tamsulosin, one study assessed the use of silodosin, and one study compared silodosin to tamsulosin and control. Six of the studies assessed the stone expulsion rate, and four (10, 12, 14, 17) of them found a statistically significant increase in expulsion rate while two studies failed to do so (10, 17). Six studies assessed the stone expulsion time, and five studies found a statistically decreased expulsion time compared to control (10, 12, 14, 16, 17), while one failed to do so (10).

One study assessed the use of tamsulosin after initial SWL episodes for stones located in the renal pelvis, and found no significant increase in stone expulsion rate or decrease in stone expulsion time between groups (16). Two studies assessed the use of alpha-blockers regardless of stone location (11, 13). One study using doxazosin after initial SWL episode, and found no change in stone expulsion time, stone expulsion rate, and number of repeat SWL episodes (12). One study found tamsulosin to be effective in increasing stone expulsion rate compared to control, however found smaller stones and stones in the UVJ relative to the UPJ were more likely to pass (14).

### Risk of bias and quality assessments in selected studies

The results of the risk of bias and quality assessments are presented in Tables 1, 2, respectively. Four studies presented with low risk of selection bias due to random sequence generation (11, 16–18) and three studies had unclear risk (10, 13–15). Five studies presented with high risk of selection bias due to allocation concealment (10, 11, 13, 15, 18), one study had unclear risk (16), and one study had low risk of bias (17). Five studies had high risk for performance bias due to blinding of the personnel (10, 11, 13, 15, 16), 2 studies had low risk of bias (17, 18). Five studies had a high risk of detection bias due to blinding of self-reported outcomes (10, 11, 13, 15, 16), two studies had low risk (13, 16, 17). Four studies had high risk of bias due to blinding of reaction times outcome (9–11, 15), three studies had low risk (13, 16, 17). Three studies had low risk of attrition bias due to incomplete outcome data (10, 13, 18), three studies had high risk (11, 16, 17), and one study had unclear risk (15). Six studies had unclear risk of reporting bias due to selective reporting (11, 13, 15–18), one study had low risk (10). Five studies had low risk of other biases (10, 15–18), and two studies had high risk (11, 13). Of the two cohort studies, one study had a full score of 8/8 (14), and the other study had a score of 5/8 (12).

**Table 1:** Risk of bias assessment of included studies.

	Aldaqadossi et al.	Shahat et al.	Elgalaly et al.	Soliman et al.	Mokhless et al.	Erturhan et al.	Aydogu et al.
Random sequence generation (selection bias)	Unclear risk	Low risk	Low risk	Low risk	Unclear risk	Low risk	Unclear risk
Allocation concealment (selection bias)	High risk	Unclear risk	Low risk	High risk	High risk	High risk	High risk
Blinding of participants and personnel (performance bias)	High risk	High risk	Low risk	Low risk	High risk	High risk	High risk
Blinding of outcome assessment (detection bias) self-reported outcomes	High risk	High risk	Low risk	Low risk	High risk	High risk	High risk
Blinding of outcome assessment (detection bias) reaction time	High risk	High risk	Low risk	Low risk	High risk	High risk	High risk
Incomplete outcome data (attrition bias)	Unclear risk	High risk	High risk	Low risk	Low risk	High risk	Low risk
Selective reporting (reporting bias)	Unclear risk	Unclear risk	Unclear risk	Unclear risk	Unclear risk	Unclear risk	Low risk
Other bias	Low risk	Low risk	Low risk	Low risk	High risk	High risk	Low risk

**Table 2:** Quality assessment of cohort studies.

	Selection Bias				Outcomes			Comparibility	Total
	Representativeness of the Cohort	Selection of Non-Exposed Cohort	Ascertainment of Exposure	Outcome not present at the start of the study	Assessment of Outcomes	Length of Follow-up	Adequacy of Follow-up		
Telli et al.	+	+	+	+	+	+	+	+	++++++++
Tasian et al.			+	+	+	+	+		+++++

## Discussion

There is a growing body of literature on the use of alpha blockers for children with kidney stones. While the use of alpha blockers is generally considered safe in children, their effectiveness in managing kidney stones is still unclear, and more research is needed to determine their optimal use. This systematic review synthesizes the findings of nine studies assessing the use of alpha blockers (doxazosin, tamsulosin, and silodosin) in the treatment of pediatric kidney stones. Our findings suggest that alpha blockers are a promising adjunct for the management of pediatric stones, though the heterogeneity of the studies and data generated by the included studies must be acknowledged and may weaken these conclusions.

Alpha blockers have gained interest as potential management options for pediatric kidney stones due to their safety, cost-effectiveness, and ease to administer all while being effective. By comparison, surgical modalities are costly, require anesthesia, and are more burdensome when compared to medical expulsive therapy. Alpha blockers have shown promising results in adults stone management (19). In pediatric patients, McGhee et al. found higher ureteroscopic access rates with tamsulosin pre-treatment for 1 week (20), reinforcing a role for alpha blockers in surgical interventions if medical expulsive therapy fails. While there is some evidence to suggest that alpha blockers may be effective in managing kidney stones in children, available studies are limited by various methodological concerns, such as small sample sizes and lack of blinding.

In our review, we found that reported outcomes were most promising for the use of tamsulosin. Most of the included studies demonstrated statistically significant improvement in stone parameters, including decreased stone passage time and increased stone passage rate when compared to control. The one study that did not find such difference assessed the use of tamsulosin after SWL, and found no difference among treatment and control groups, indicating that the use of alpha blockers may not be as effective after SWL as when they are given alone.

Despite the fact use of silodosin for kidney stones is off-label even in adult patients, our review suggests it may be effective in the pediatric population, albeit this agent was the least studied of the 3 alpha blockers included our review. One study found silodosin superior to tamsulosin in both decreasing stone passage time and increasing stone passage rate (18). In the other included study, there was no statistically significant difference in stone passage rate between silodosin and control, but there was a decreased stone passage time in the treatment group (17). Studies in the adult population have found silodosin to be superior to tamsulosin in stone expulsion, probably due to its increased alpha selectivity (21). Further comparative study would further clarify these results but may be hindered by the off-label designation of silodosin.

We found the data were varied for doxazosin. Although most studies of doxazosin revealed decreased stone passage time, two of the three included studies found no statistically significant difference in the stone passage rate between the treatment and control group. It is also worth noting that stones passage rate was higher for younger patients and smaller stones.

Pain outcomes appeared to be improved with use of alpha blockers. While not all studies measured these parameters, many included studies noted a significantly decreased mean number of pain episodes in the alpha blocker group compared to the control, with some studies noting a significant decrease in need for analgesics in the treatment group. This suggests a dual benefit of alpha blocker use in stone management, for both stone passage and pain during the stone episode.

In general, the various alpha blockers included in this review were well tolerated, and with a desirable adverse effect profile. A recent meta-analysis by Sun et al. validates the findings of this present study. The meta-analysis included 7 clinical trials and cohort studies. It reports the efficacy of alpha blockers in kidney stones while maintaining a high safety profile with minimal adverse events (22).

Similar results have been noted in the adult population. A systematic review by Seitz et al. found higher rates and faster expulsion times with alpha blockers compared to control. However, it notes that trials assessing the use of alpha blockers after SWL have shown promising results in the adult population. One study included in this present review assesses the use of tamsulosin after SWL, however this study failed to find any statistically significant results (23). As seen in the present review, prior reviews of adult literature also highlight some heterogeneity in the data on alpha blockers use in the adult population (24). However, when compared to pediatric literature, there is a sizeable body of evidence to support the efficacy of alpha blockers for distal ureteral stones in adults (25). We have evidence both in our review and others that this statement holds true for children as well (6, 10–18).

While there is growing evidence that alpha blockers are useful adjuncts therapy for medical management of stone passage in children, in a clinical real-world setting alpha blockers remain underutilized (26). Prior studies suggests that involvement of a urologist during an acute stone episode increases likelihood that a pediatric patient will receive an alpha blocker as part of a presenting treatment plan (27) We surmise that lack of familiarity with dosing and discomfort with administration for younger patients may contribute to its underuse.

In our risk of bias assessment, we found high risk in several domains. We surmise that this is primarily due to design limitation, with some studies utilizing inadequate randomization or blinding, and secondarily due to reporting limitations, as some studies did not adequately their experimental methods to ensure low risk of bias. Limitations cited by each study included small study size (10, 11, 13, 17), lack of placebo group, randomization and/or blinding (10–12, 16) challenges with objective evaluation of analgesic requirement (10, 12), the retrospective nature of included cohort studies (12, 14), heterogeneity of variables and interventions, off-label use of interventions (18), and imaging-related limitations (14, 17). Overall, we found that the heterogeneity of findings on the efficacy of alpha blockers within our review may be attributed in part due to these limitations.

We also acknowledge possible limitations in the design of this review. First, additional databases could have been included in our initial search. Second, the noted design limitations for some studies likely also limit our review. Third, our choice of broad inclusion for studies of stones in all locations, not just ureteral, may dilute the strength of our conclusions. However, to our knowledge this is the first review to include stones in all locations in a pediatric population.

More studies are needed to assess the use of alpha blockers in pediatric kidney stones and would ideally report larger sample sizes from multi-institutional cohorts. While we note that our conclusions would be strengthened by additional high-quality studies on this topic, our review suggests that alpha blockers are a useful and safe option for the non-operative management of pediatric nephrolithiasis. Tamsulosin and silodosin appear to be the best-studied agents, with data showing improvement in stone passage and pain outcomes for stones in the distal ureter.

## Conclusion

In this systematic review, we found that alpha blockers are effective in managing kidney stones in a pediatric population. There is support for the use of tamsulosin in the setting of distal ureteral stones, but our results suggest efficacy with use of doxazosin and silodosin as well. Results were mixed in the use of alpha blockers after SWL. While study design was heterogenous and may have introduced higher risk of bias, several studies demonstrated superiority in the use of alpha blockers compared to watchful waiting. Continued evaluation of alpha-blocker treatment in this population through high-quality, multi-center trials is recommended.
